# Videogames for academic wellbeing: a biopsychosocial health psychology framework for university counseling and mental health promotion

**DOI:** 10.3389/fpsyg.2026.1935433

**Published:** 2026-08-26

**Authors:** Eugenio De Gregorio, Ilaria De Laurentiis

**Affiliations:** Department of Humanities, Link Campus University, Rome, Italy

**Keywords:** academic wellbeing, digital mental health, health psychology, motivation, psychological counseling, university, videogames

## Abstract

In recent years, videogames have attracted growing attention not only for their recreational value, but also for their potential to foster personal and social identity, enhance cognitive functioning, and promote psychological wellbeing. Consistent with a biopsychosocial perspective on health, this paper conceptualizes gaming as a psychological, behavioral, and social resource that can be purposefully integrated into health-promotion practice, and explores its use within care contexts, with a particular focus on university students. In 2025, Link Campus University in Rome launched a psychological support service funded by the Wave Pro-ben project (Project ID: PROBEN_0000007), supported by the Italian Ministry of University. The service targets students enrolled in degree programmes, PhDs, and master's courses, addressing emotional, relational, academic, and adaptation-related challenges. Counseling initiatives promote psychological wellbeing, prevent distress, and foster personal growth, thereby reducing dropout risk and supporting academic success. Activities include individual sessions, support groups, workshops, and psychoeducational interventions, offered confidentially and free of charge—a design choice intended to reduce barriers of access and stigma. The distinctive element of the service is the integration of videogames as tools for reflection and change. Traditional counseling has been complemented by structured gameplay, drawing on Video Game Therapy^®^ (VGT), developed by Francesco Bocci. VGT^®^ is conceptually intended to create opportunities for emotional recognition and regulation, cognitive engagement, agency, and social-skill development; however, evidence specifically evaluating VGT^®^ remains preliminary. Research shows that, while excessive gaming carries risks—such as isolation, anxiety, and problematic use—balanced and intentional play can provide meaningful benefits, stimulating flow states in which individuals process emotions, revisit past experiences, and develop adaptive coping strategies. By integrating games into counseling, the approach also engages students who may avoid traditional support due to stigma or skepticism, thus contributing to more equitable access to psychological care in higher education. As a conceptual and programmatic Community Case Study, the paper does not present effectiveness data. Instead, it describes the theoretical rationale, institutional context and key operational elements of the model, distinguishes the broader evidence on videogaming and wellbeing from the still preliminary evidence specific to VGT^®^, and outlines a framework for prospective empirical evaluation.

## 1 Introduction

Health Psychology seeks to understand how psychological, biobehavioural, and environmental factors jointly influence mental and physical health, wellbeing, and illness. Within this biopsychosocial framework, leisure practices such as videogaming are increasingly recognized as behaviors that can shape—for better or worse—emotional regulation, cognition, and social connection. Over the past two decades, videogames have accordingly evolved from being perceived as mere entertainment to becoming a subject of serious scientific inquiry in relation to psychological wellbeing. A growing body of research suggests that, when engaged with in moderation and in appropriate contexts, videogames can provide significant benefits across emotional, social, and cognitive domains, with effects varying by age group and type of gameplay.

Johnson et al. (2013) conducted one of the earliest comprehensive reviews on videogames and wellbeing, focusing on adolescents and young adults (12–25 years). Their findings challenged the dominant narrative of games as detrimental, highlighting instead how play can foster emotional stability, stress reduction, and positive mood regulation. Moderate gaming was found to correlate with higher self-esteem, optimism, resilience, and vitality. Importantly, multiplayer and online role-playing games were shown to cultivate social capital and supportive relationships, sometimes even surpassing offline friendships in depth and emotional openness. These outcomes indicate that videogames can be tools of self-expression and identity exploration, particularly for youth negotiating the transition from adolescence to adulthood.

Kolivand et al. (2022) broadened the scope by reviewing decades of research on the impact of games across the lifespan. While acknowledging concerns around violent content and excessive play, the review documented significant cognitive and psychosocial benefits. Adolescents were shown to gain motivation, problem-solving abilities, and digital literacy, which could translate into educational contexts. For adults, games served as stress relievers and social connectors, particularly through online communities. Although methodological inconsistencies remain in the literature, the review emphasized that the developmental stage at which individuals engage with games strongly influences whether the outcomes are adaptive or maladaptive.

Zhao et al. (2024) focused specifically on active video games (AVGs), or exergames, and their effects on college students' mental health. Synthesizing 17 randomized controlled trials, the review found robust evidence that AVGs reduce stress, anxiety, and depression, while simultaneously increasing happiness, psychological satisfaction, and sleep quality. Beyond mental health, these games boosted motivation for physical activity and improved attitudes toward exercise—critical given the global epidemic of sedentary lifestyles among young adults. AVGs thus bridge the gap between digital entertainment and physical movement, transforming “screen time” into a health-promoting activity that engages biological, psychological, and social dimensions of health simultaneously. The authors concluded that carefully designed AVGs could function as scalable interventions for university populations at high risk of poor mental health outcomes.

These studies converge on the view that videogames are not a monolithic influence but rather a diverse medium whose associations with psychological wellbeing depend on context, content, motivation, and patterns of use. Rather than being understood exclusively as passive distractions, videogames may, under specific conditions, function as interactive environments associated with resilience, social connectedness, and dimensions of psychological wellbeing.

## 2 Background and rationale

University students represent a population of particular concern for community health psychology: emerging adulthood is a period of heightened vulnerability to psychological distress, and academic environments add specific stressors related to performance, adjustment, and identity. International literature now appears to support the link between videogames, psychological wellbeing, and academic performance (Capinpin et al., 2022). The studies reviewed primarily concern the direct relationship between videogames—as a method and tool—and psychological wellbeing, as well as the potential to extend this approach beyond youth populations. However, to the best of our knowledge, no initiatives have yet been described in the literature that implement a psychological support service for university students through the structured use of videogames. The present paper aims to address this gap by describing the conceptual rationale and programmatic elements of such a service.

In a recent contribution, Fritzon et al. (2024) note that playing videogames can have positive effects on mental health, particularly for young people. Moderate gaming has been linked to improvements in mood, stress reduction, and a stronger sense of social connection when games are played with others; further positive effects concern lower levels of stress and increased creativity. Videogames can provide distraction from everyday worries, foster resilience, and create opportunities for achievement and mastery, which in turn can boost self-esteem. Other surveys indicate that videogames can effectively reduce stress and help foster social connections, including online ones, and certain games can enhance specific cognitive abilities such as visuo-spatial navigation and problem-solving.

Games and technologies can also specifically target mental health challenges—such as social anxiety or phobias—address ADHD symptoms, and boost motivation and performance. At the same time, concerns persist regarding potential long-term effects, especially declines in offline social interaction, engagement in physical activity, academic outcomes, and health-related habits such as sleep and diet. The literature therefore stresses the importance of balance: while moderate play supports psychological wellbeing, excessive gaming may lead to negative consequences such as sleep problems or social withdrawal. Overall, the findings suggest that videogames, when integrated in a healthy and balanced way, can be a valuable tool to enhance psychological wellbeing and life satisfaction.

Positive effects have also been documented in the adult population, where videogames have been used to support cognitive functioning and emotional training. The systematic review by Pallavicini et al. (2018) highlights how videogames can play a significant role in enhancing both cognitive abilities and emotional wellbeing in healthy adults. Across the studies examined, videogames improved several mental functions, including processing speed, memory, multitasking, and spatial skills: action and racing games consistently boosted reaction times, puzzle and strategy games enhanced cognitive flexibility, and adventure games produced measurable changes in brain structure linked to memory and navigation. Beyond cognitive performance, videogames also proved effective in supporting emotional regulation: some games helped reduce stress and anxiety, while others improved mood and promoted positive emotions. Exergames in particular were associated with increased enjoyment and relaxation, while specially designed training games showed benefits in reducing attentional biases linked to anxiety. In our view, these positive effects may extend beyond strictly cognitive and emotional dimensions to aspects more typically linked to motivation, self-realization, problem solving, decision making, and life satisfaction in academic settings.

The use of videogames has also been related directly to student wellbeing. Johannes et al. (2021) investigated the relationship between videogaming and mental health outcomes, addressing long-standing concerns that extensive playtime might harm psychological wellbeing. Noting that much prior research relied on self-reported playtime—shown to be unreliable and prone to recall bias—the authors partnered with Electronic Arts and Nintendo of America to obtain telemetry data (objective logs of in-game activity) and combined these records with survey measures of wellbeing and motivational experiences. The study surveyed players of *Plants vs. Zombies: Battle for Neighborville* and *Animal Crossing: New Horizons* (total N ≈ 3,200 with matched telemetry), assessing affective wellbeing with the Scale of Positive and Negative Experiences (SPANE) and player experiences with the Player Experience and Need Satisfaction scale (PENS). Analyses revealed a small but statistically significant positive correlation between objectively measured playtime and wellbeing. The motivational context of play emerged as a key independent predictor: feelings of autonomy, competence, relatedness, and intrinsic enjoyment during play were positively associated with wellbeing, whereas extrinsic motivations (e.g., playing out of obligation or escapism) were negatively correlated. The observed effect sizes were modest, suggesting that while gaming alone is unlikely to produce clinically significant changes in wellbeing, its role as a source of daily enjoyment and social interaction may have cumulative benefits over time. Methodologically, the study also demonstrates that collaborations between academia and industry can yield transparent, high-quality data for evidence-based practice.

von der Heiden et al. (2019) present a large-scale, cross-sectional investigation into the psychological correlates of videogame use, moving beyond simple playtime to examine problematic gaming behavior, motivations for playing, and preferred genres in relation to psychopathology, coping, affectivity, social relationships, and self-perceptions. The study surveyed 2,734 German participants (mean age 23.06 years) using the AICA-S (based on DSM-5 Internet Gaming Disorder criteria) and validated measures of psychopathology (SCL-K-9), coping (Brief COPE), affect (PANAS), self-esteem, self-efficacy, social support, loneliness, and shyness. A central finding was a medium-sized negative association between psychological wellbeing and problematic videogame use. Higher AICA-S scores were associated with:

- elevated psychological symptoms (e.g., depression, anxiety, hostility);
- maladaptive coping strategies such as self-blame, denial, behavioral disengagement, and substance use, together with lower adaptive coping (active coping, planning, positive reframing);
- higher negative affect both generally and during gameplay, but also increased positive affect during play, indicating gaming's dual role as a source of enjoyment and distress;
- social-emotional challenges, including shyness, loneliness, and preference for solitude;
- lower life satisfaction, reduced self-esteem, lower self-efficacy, weaker perceived offline social support, and poorer academic performance.

Interestingly, problematic gamers tended to have fewer offline friendships but more online connections, emphasizing gaming's role in social compensation and digital community building. Motivational analyses revealed that reasons for playing were strongly predictive of psychological functioning: escape-oriented motives correlated with higher psychopathology, negative affectivity, low self-esteem, loneliness, poorer academic achievement, and maladaptive coping; socially driven motives were linked to stronger positive affect during gameplay and extensive online networks, suggesting that gaming can fulfill unmet social needs; curiosity, imagination, and narrative engagement were tied to positive in-game experiences with weaker associations with negative functioning. These findings highlight the paradoxical interplay of psychological vulnerabilities and gaming's reinforcing qualities—and, for intervention design, the importance of working on the quality and motivational context of play rather than its mere quantity.

In clinical settings, the Video Game Therapy^®^ model has been proposed (see next section); it provides conceptual support to the proposal presented here. The key element of this paper, however, is the innovative use of videogames in university contexts to foster and support student wellbeing in a non-clinical, non-psychotherapeutic setting—an intervention model explicitly targeting psychological wellbeing, flourishing, meaning, and social connection.

## 3 Description of the case

The service described here was developed within the national PRO-BEN programme, funded by the Italian Ministry of University and Research to promote the psychophysical wellbeing of the university student population. Link Campus University participates as a partner institution in the Lazio 1 partnership, named “Wave”, coordinated by Sapienza Università di Roma as lead institution, together with LUMSA University, the University of Tuscia, the University of Rome “Foro Italico”, the University of International Studies of Rome (UNINT), the Rome Academy of Fine Arts and the “Silvio D'Amico” National Academy of Dramatic Art; a parallel partnership (Lazio 2) is coordinated by the University of Rome Tor Vergata. Within this framework each partner designs and delivers its own initiatives, while the partnership as a whole pursues a shared regional strategy for the promotion of student psychological wellbeing. This structure is relevant for international readers because it situates the present case within a regionally coordinated and nationally funded framework rather than an isolated institutional experiment. The videogame-based component described in this paper was designed and implemented by Link Campus University as its own contribution to the partnership (Project ID: PROBEN_0000007).

This Community Case Study describes the development and early implementation of a university-based counseling model rather than reporting the findings of an effectiveness trial. Accordingly, the case is presented in terms of its institutional context, theoretical rationale, operational characteristics, implementation challenges, and prospective evaluation framework.

The proposal concerns the use of videogames as a psychological support and counseling tool for university students. The project—currently being implemented at Link Campus University in Rome within the Wave Pro-ben project funded by the Italian Ministry of University—applies Video Game Therapy^®^ (VGT) to a context that is not purely clinical or therapeutic, with the aim of increasing students' wellbeing and intervening early on emerging forms of distress, ahead of the risk of academic dropout. The service is offered confidentially and free of charge to students enrolled in degree programmes, PhDs, and master's courses; this universal, no-cost access is itself a programmatic choice consistent with the goal of reducing inequalities in access to psychological care (in line with SDG 3, Good Health and Wellbeing, and SDG 10, Reduced Inequalities).

According to Bocci and Girelli (2024), Video Game Therapy^®^ is an innovative, integrated psychological approach that uses commercial videogames within a structured setting to foster emotional regulation, cognitive balance, and personal growth. Unlike traditional talk-based approaches, VGT leverages the immersive and interactive qualities of videogames to help individuals feel safe, engaged, and open to exploring aspects of themselves that may otherwise remain hidden. The authors claim that one of the primary benefits of VGT is its ability to create a secure environment where individuals can express emotions freely and reduce psychological defenses. This setting encourages authentic self-exploration, insight, and reflection on personal patterns and life choices. Through gameplay, individuals enter a state of flow, in which challenges and skills are balanced, fostering resilience, concentration, and emotional stability. The process also promotes the development of life skills and soft skills, such as problem solving, decision making, emotional awareness, cooperation, and empathy. By confronting challenges in a virtual world—where failure does not carry real-life consequences—individuals can practice coping strategies, build confidence, and translate these experiences into everyday life.

Another central aspect of VGT is its connection with Compassion-Focused Therapy (CFT; Gilbert, 2009), which constitutes one of the main general frames of the approach. CFT trains individuals to access their “soothing system”, a neurophysiological state of safety and calmness associated with feelings of affiliation, kindness, and acceptance. This helps counteract self-criticism, anxiety, and rigid patterns of judgement, while encouraging emotional regulation and social connectedness—all aspects that may be crucial for adapting to academic environments. Furthermore, VGT functions as an experiential learning model: participants are not passive recipients of advice but active protagonists of their own journey, experimenting with new behaviors, reflecting on outcomes, and gradually integrating insights into their personal lives. This experiential process facilitates resilience, flexibility, and lasting change.

Preliminary case-based accounts suggest that VGT^®^ may facilitate engagement among individuals experiencing psychological or psychiatric difficulties and may provide opportunities to explore emotional recognition, regulation, and coping strategies. These observations should be regarded as exploratory and hypothesis-generating rather than as evidence of established clinical effectiveness.

In broader applications, the method has also been proposed for educational and community contexts, where its potential contribution to wellbeing, digital awareness, and personal empowerment warrants systematic empirical investigation.

In brief, Bocci and Girelli (2024) identify the following key benefits of VGT^®^:

- it provides a safe and non-judgemental environment that reduces defenses;
- it facilitates authentic emotional expression and insight into the self;
- it induces flow states, enhancing concentration, resilience, and balance;
- it develops life skills and soft skills (problem solving, decision making, empathy, cooperation);
- it strengthens emotional regulation and reduces self-criticism through compassion-focused strategies;
- it encourages experiential learning, whereby participants actively practice and internalize new coping strategies;
- it is applicable across different age groups and contexts, from children to adults, in clinical, educational, and preventive settings.

Within the counseling service, working on the self through VGT^®^ is conceptualized as a training process in which students learn to manage emotions and difficult situations more effectively when they recur; to develop compassion and understanding for themselves and others; to recognize the situations that trigger dysfunctional behavioral patterns; and to set healthier boundaries through the continuous training of life skills. The narrative structure of gameplay—the “hero's journey”—offers students a metaphorical space in which to clarify goals, acknowledge choices, and experience agency: as in a book, life is made up of different chapters, and in the videogame students can rehearse decisions and take note of their consequences without real-life costs.

Underlying healthy boundaries is self-awareness: through training supported by VGT^®^, students can develop the ability to recognize who they are, what they feel, what they need (in terms of life skills and social connection), and where they wish to invest their efforts. On this basis they can define boundaries within which they feel part of their environment—not mere spectators, but protagonists of their own lives, including in the important choices concerning their positioning toward the university. In this sense, the intervention targets not only symptom prevention but positive dimensions of functioning—flourishing, meaning, and social connection—consistent with a holistic, biopsychosocial understanding of health.

Beginning in May 2025, through the Wave Pro-ben project, Link Campus University officially incorporated videogames into its counseling service, complementing individual sessions, support groups, workshops, and psychoeducational interventions. Rising demand for the service underscores the importance of such initiatives in higher education. By integrating games into counseling, the approach also engages students who may avoid traditional psychological support due to stigma or skepticism, promoting a more inclusive and engaging experience and expanding opportunities for psychological intervention.

## 4 Methodological aspects

Within the university counseling service, videogame-based activities are not conceived as a stand-alone intervention replacing conventional psychological counseling, but as an additional resource that may be integrated into the counseling process when considered appropriate to the student's needs, characteristics, and objectives. Access to the service is available to the university population described above. The appropriateness of incorporating videogames is considered within the counseling process rather than assumed for all service users.

Access to the service was universal within the university population: no eligibility or exclusion criteria were applied at intake, consistent with the free and confidential design described above. Decisions regarding referral to external services were therefore reached through clinical judgement during the counseling process rather than through pre-defined exclusion criteria or a standardized screening instrument.

The service model envisaged two to three counseling sessions per student. Because the initiative was established as a student-support service rather than as a research protocol, the number and duration of the sessions actually delivered were not systematically recorded; the figure of two to three sessions should therefore be understood as a parameter of the service design rather than as an observed average.

Game-integrated sessions are conducted by psychologists who, in addition to their professional qualification, have completed a specific training course conferring the qualification of Expert in Video Game Therapy^®^ delivered by videogametherapy.it

Game selection is guided by the objectives emerging during counseling, the student's previous gaming experience and preferences, and the psychological processes that the game may help to explore. Commercial videogames are therefore not conceptualized as having therapeutic properties in themselves; their relevance derives from how gameplay is embedded in a professionally facilitated reflective process. Gameplay may provide material for exploring decision making, emotional responses, cooperation, frustration tolerance, agency, or narrative identification. Reflective discussion and debriefing connect experiences occurring within the game with situations, emotions, and relational patterns in the student's every day and academic life.

**Figure 1:**
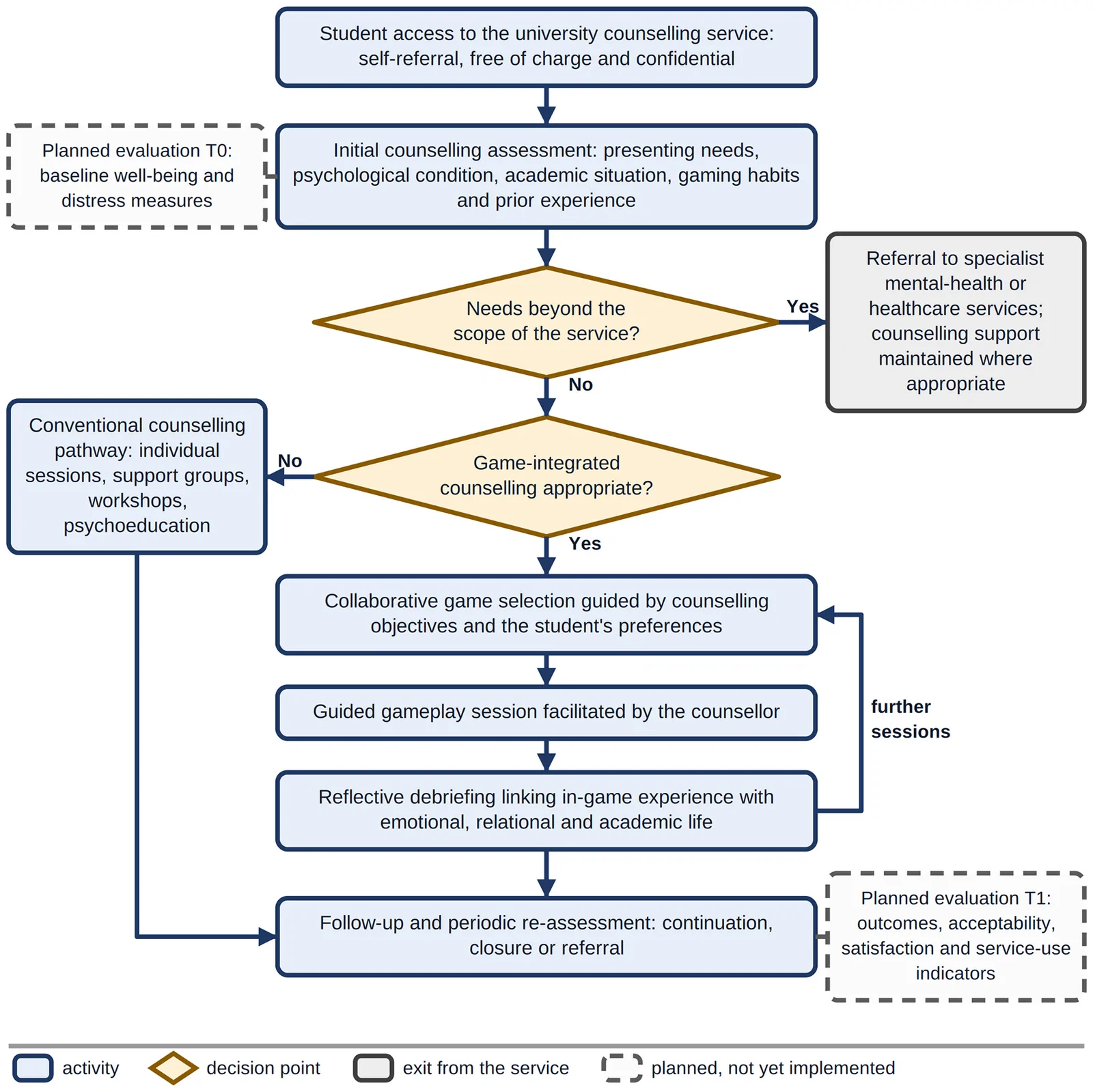
Student pathway through the university counseling service. The pathway represents the conceptual operational sequence described in the manuscript. Game-integrated counseling is not assumed to be appropriate for every student.

Where assessment indicates needs exceeding the scope of university counseling, referral to appropriate healthcare services should be considered. In particular, problematic or escape-driven gaming requires careful assessment rather than automatic encouragement of further gaming. The model therefore treats game integration as conditional, individualized, and subordinate to the broader aims and professional boundaries of counseling (Figure 1).

Table 1 matches and clearly distinguishes the role of video games in a non-clinical preventive-promotive context at a university with the role of video games in a clinical therapy context (conventional VGT^®^) for patients with severe psychiatric disorders, even.

**Table 1:** Conceptual distinction between clinical VGT^®^ and the university game-integrated counseling model.

Dimension	Clinical VGT^®^	University counseling model described here
Setting	Clinical/therapeutic	University/community
Primary orientation	Therapeutic	Preventive and health-promoting
Target population	Individuals presenting clinical or psychological difficulties	University students seeking psychological support
Role of videogames	Component of a structured therapeutic approach	Additional resource integrated into counseling
Primary aims	Therapeutic change and management of psychological difficulties	Wellbeing, self-awareness, coping, academic adjustment and prevention of distress
Clinical diagnosis	May be relevant to treatment planning	Not a prerequisite for accessing the service
Game selection	Related to therapeutic formulation and objectives	Related to counseling goals, student characteristics and gaming preferences
Professional context	Clinical/psychotherapeutic relationship	Psychological counseling and health-promotion setting
Evidence base	Emerging and currently limited specifically for VGT^®^	Model currently undergoing implementation; effectiveness yet to be established
Expected evaluation	Clinical outcomes	Wellbeing, distress, feasibility, acceptability, engagement and academic-related outcomes

## 5 Implementation and evaluation considerations

The implementation of videogame-based counseling raises practical and ethical challenges. Students differ substantially in familiarity with, interest in, and attitudes toward videogames; game-based activities should therefore not be assumed to be equally acceptable or appropriate for all service users. Individual gaming preferences, previous experiences, accessibility needs, cultural meanings associated with gaming, and possible technological barriers should inform intervention decisions. Professional competence is also relevant: practitioners require counseling skills together with sufficient understanding of videogame environments and of the rationale for their reflective use. Ethical considerations include informed consent, privacy and data protection where online or networked games are used, appropriate boundaries between recreational and counseling activities, and continuing assessment of whether game-based methods remain consistent with the student's needs and best interests.

Although the service has been operational since May 2025, it was established primarily as a student-support service rather than as a research study. Consequently, systematic baseline, outcome, satisfaction, and qualitative-feedback measures suitable for research purposes were not embedded in the initial implementation phase. Such data are therefore not available for the present manuscript, and administrative information should not be retrospectively reconstructed or interpreted as evidence of effectiveness. This represents an important limitation of the present Community Case Study and directly informs the prospective evaluation framework proposed below.

## 6 Discussion

The literature reviewed provides converging evidence that videogames, when integrated into daily life with balance and intentionality, can positively affect psychological wellbeing across the lifespan. Far from being merely recreational tools, videogames have increasingly been recognized as interactive ecosystems capable of fostering resilience, enhancing social connectedness, and promoting cognitive and emotional growth (Johnson et al., 2013; Kolivand et al., 2022; Zhao et al., 2024). At the same time, many of the sources we have drawn on are reviews in which the specific link between videogames and psychological wellbeing has been examined in only a limited number of primary studies; the evidence base, while promising, remains uneven.

Across age groups, the benefits of gaming manifest in different domains. For adolescents, games provide avenues for identity exploration, self-expression, and the development of social capital. For adults, they function as stress relievers, cognitive enhancers, and sources of meaningful social interaction. For young adults and university students in particular, active video games appear to bridge the gap between digital entertainment and health-promoting behaviors, reducing anxiety and depression while supporting motivation for physical activity. Collectively, these findings suggest that videogames may have potential as resources for supporting psychological functioning when used in moderation and within appropriately structured context.

Emerging approaches such as Video Game Therapy^®^ take this potential a step further by integrating videogames into formal psychological support systems. Unlike traditional modalities, VGT leverages the immersive and interactive qualities of commercial games to create safe environments for emotional expression, resilience-building, and personal growth (Bocci and Girelli, 2024). By inducing flow states, fostering compassion-focused processes, and promoting experiential learning, VGT can help individuals regulate emotions, practice problem-solving, and strengthen life skills in ways that traditional interventions may not.

The application of VGT in university contexts represents a particularly innovative development for health psychology. University students face unique challenges—academic stress, social adjustment, and dropout risk—all closely linked to psychological wellbeing. Using videogames not only as therapeutic tools but as preventive, supportive interventions within higher education opens new directions for promoting student mental health at the community level: the university is here understood as a community setting in which health promotion can be embedded. By offering structured opportunities for emotional regulation, self-awareness, and resilience training, videogames can help students navigate transitional periods while reducing vulnerability to psychological distress. Because the medium is familiar and non-stigmatizing for this population, the model may also reach students who would not otherwise access counseling, contributing to more equitable service uptake.

VGT^®^ is theoretically proposed to draw on flow experiences, compassion-focused processes, and experiential learning to create opportunities for emotional reflection, problem-solving, and life-skill development. Whether these mechanisms produce outcomes beyond those achievable through conventional counseling remains an empirical question requiring direct investigation.

Nonetheless, the field must proceed cautiously. As highlighted by recent studies (Johannes et al., 2021; von der Heiden et al., 2019), the benefits of gaming are contingent on motivational context, play style, and balance. Excessive or escape-driven gaming remains associated with negative outcomes such as social withdrawal, reduced academic performance, and maladaptive coping. The key therefore lies not in the quantity of play but in its quality and integration into healthy routines—a principle that the counseling setting itself is designed to safeguard, through professional facilitation and reflective debriefing of gameplay.

Theoretical perspectives such as compassion-focused approaches (Gilbert, 2009) and polyvagal theory (Porges, 2011) may provide hypotheses regarding processes of perceived safety, affiliation, self-compassion, and emotional regulation that could be explored in future research on game-based counseling. At present, however, these processes should be regarded as theoretical propositions rather than established mechanisms of action specific to VGT^®^.

## 7 Lesson learned and recommendations

The early implementation of the service highlights several lessons relevant to universities considering the integration of videogames into psychological support programmes. First, videogames should be understood as optional and flexible resources within counseling rather than as universally appropriate tools or substitutes for conventional psychological support. Their use should be guided by students' needs, preferences, gaming experiences, and counseling objectives.

Second, implementation requires a clear distinction between preventive-promotive university counseling and clinical videogame-based interventions. The model described here is situated within a community health-promotion framework and does not assume that evidence concerning videogaming in general constitutes evidence of effectiveness for VGT^®^ or for the specific university intervention.

Third, game-integrated counseling requires explicit operational attention to assessment, appropriateness, game selection, professional competence, reflective debriefing, referral pathways, privacy, accessibility, cultural factors, and problematic gaming.

Future work should evaluate the service through a multidimensional framework encompassing effectiveness, feasibility, acceptability, and implementation outcomes. At the individual level, prospective pre–post assessments may examine psychological wellbeing, psychological distress, emotional regulation, academic engagement, perceived social connectedness, and dropout intention. At the service level, implementation indicators may include uptake, attendance, completion, retention, reasons for discontinuation, and patterns of engagement with game-integrated vs. conventional counseling. Acceptability and feasibility should be assessed through participant satisfaction, perceived usefulness, and qualitative accounts of students' experiences. Longer-term follow-up may explore whether changes in wellbeing, coping, academic adjustment, and help-seeking behavior are maintained over time. Where feasible, future studies should compare game-integrated counseling with standard counseling formats.

Given the early stage of systematic evaluation, an initial feasibility study may be more appropriate than a definitive effectiveness trial. This would allow the intervention protocol, outcome measures, recruitment procedures, acceptability criteria, and referral processes to be refined before larger comparative studies are undertaken.

A central lesson from the present case is therefore that implementation and evaluation should develop concurrently. Future university programmes adopting similar approaches should incorporate baseline measures, service-utilization indicators, participant-reported experience and satisfaction, qualitative feedback, and longitudinal outcome assessment from the beginning of service delivery. This would allow innovation in service design to be accompanied by progressive accumulation of empirical evidence.

In conclusion, videogames may be considered not only as a form of entertainment but as potential resources within psychological support and education. In the university setting, where students navigate developmental transitions and high performance demands, their structured application may represent a promising preventive strategy for supporting wellbeing and potentially reducing academic attrition. The scientific value of this model will depend on transparent operationalization and prospective empirical evaluation.

## Data Availability

The original contributions presented in the study are included in the article/supplementary material, further inquiries can be directed to the corresponding author/s.
